# Caring, sharing, preparing and declaring: how do hospices support prisons to provide palliative and end of life care? A qualitative descriptive study using telephone interviews

**DOI:** 10.1177/0269216320979194

**Published:** 2020-12-10

**Authors:** Chris McParland, Bridget Johnston

**Affiliations:** 1 School of Medicine, Dentistry and Nursing, University of Glasgow, Glasgow, UK; 2 NHS Greater Glasgow and Clyde, Glasgow, UK

**Keywords:** Qualitative research, prisons, prisoners, palliative care, hospices, hospice care

## Abstract

**Background:**

Older adults in prison have complex healthcare needs, and many will need palliative care before their sentence ends. Compared with prison-based hospices, little is known about the role played by community-based hospices in providing palliative care to people in prison

**Aim:**

To describe the roles Scottish hospices have adopted to support prisons to provide palliative care, and to discuss the international relevance of these findings in addressing the knowledge gap around community hospices supporting people in prison.

**Design:**

A qualitative descriptive study using semi-structured telephone interviews.

**Setting/participants:**

Representatives from all Scottish adult hospices were invited to take part in a short telephone interview and all (*N* = 17) participated.

**Results:**

Four roles were identified: *caring, sharing, preparing* and *declaring.* Most hospices employed different combinations of roles. Five (30%) hospices were engaged in *caring* (providing direct care at the prison or the hospice). Eleven (65%) hospices were engaged in *sharing* (supporting the prison by sharing knowledge and expertise). Eleven (65%) hospices were engaged in *preparing* (making preparations to support prisons). All seventeen hospices were described as *declaring* (expressing a willingness to engage with prisons to provide care).

**Conclusions:**

There are differences and similarities in the way countries provide palliative care to people in prison: many are similar to Scotland in that they do not operate prison-based hospices. Variations exist in the level of support hospices provide. Ensuring that all people in prison have equitable access to palliative care will require close collaboration between prisons and hospices on a national level.

**What is already known about the topic?**

- Older adults in prison have complex healthcare needs and many will require palliative care before the end of their sentence
- Internationally, approaches to providing palliative care in prisons include on-site hospices and support from community hospices
- 30% of Scottish hospices provide some form of support to custodial institutions, yet the nature of this support is not known

**What this paper adds?**

- This study demonstrates that the true number of hospices providing support to prisons is closer to 65% if sharing expertise with prisons is also considered a type of support.
- Hospice support ranges from those who facilitate family visits from relatives who are in prison, to those who proactively identify and provide care for people at the end of life in prison.

**Implications for practice, theory or policy**

- Prisons and hospices should work collaboratively to provide palliative care both in the prison and the hospice on a national level.
- Hospices should be adequately prepared to support people on their release from prison as well as the staff who will be caring for them.

## Introduction

In many countries, the number of older adults in prison is growing.^1–3^ People in prison suffer higher rates of many terminal illnesses than those outside prison,^4^ have high levels of multimoridity,^5^ and are twice as likely to have palliative care needs than someone of the same age and gender outside of prison.^6^ As a result, there is a growing need to understand what is currently being done to support prisons to provide palliative care to this population, and to identify sustainable practices to help meet these needs in the future.

### Ageing in prison

There is longstanding debate in relation to the way ‘older’ adults are defined in prison. A tendency exists for researchers, health services and charities to classify adults from the age of 50 as ‘older’ in prison.^7,8^ Others use 60 as the threshold at which ‘older’ is defined.^9^ Recent research from the United States of America (USA) and France indicates that people in prison develop palliative and end of life care needs several years earlier than those outside prison.^5,6^ Prost and colleagues suggest that this population already possess risk factors for poor physical and mental health prior to imprisonment, and that these are further exacerbated by being in prison.^10^ People in prison are more likely to require palliative care at a younger age than those outside of prison.

### Palliative care in prison

Approaches to delivering palliative care in prisons vary between countries, and include (but are not limited to) dedicated hospices within the prison walls, and support from specialist palliative care providers outside the prison. Much of the literature on palliative care in prisons comes from the USA,^1,11^ where prison-based hospices are comparatively common.^10^ In contrast, a recent international mapping exercise by the European Association of Palliative Care (EAPC) indicated that there were no prison hospices in Australia, Belgium, Czech Republic, France, Portugal or Slovakia.^12^ Many countries have specific mechanisms which allow people in prison to apply for compassionate release at the end of life, so that they can die outside prison. There is wide variation in the rates at which compassionate release is granted: approximately 3% of applications in the USA are successful^13^ compared with 85% in France.^12^ Yet even in countries such as France, there is evidence to suggest that this high success rate hides a large number of individuals who may be eligible for compassionate release but do not apply.^6^ Apart from a very small number of prison hospices in England and Wales,^12,14^ providing palliative care in UK prisons generally involves either transferring the individual to a hospital or hospice outside the prison, or receiving support from the hospice to care for the person while still in prison.^14^

Scotland is a geographically diverse country with a population of approximately 5.5 million, and as with many countries, this population is ageing.^15^ Approximately 8000 people who have been convicted in a court of law or are awaiting trial are housed in Scotland’s fifteen prisons.^16^ Limited data is available regarding age-related trends in the Scottish prison population; however, a 2017 report by the Scottish Prison Service indicated that the number of those over 60 had risen by a fifth in 1 year.^9^ Data from England and Wales (the only UK nations who routinely publish this data) indicates that the proportion of people aged over 50 in prison rose from 7% in 2002 to 16% in 2019.^17^ Criminal law differs between UK nations but the growth in the ageing prison population appears to be consistent.^17^

Death and declining health is a significant worry for older adults in Scotland’s prisons,^9^ a finding echoed in the international literature.^11^ Also similar to many other countries,^12^ there are no dedicated hospices inside Scottish prisons, meaning that specialist palliative care is only available from hospices or providers who serve the geographical area in which the prison is located.

Scotland is one of many countries where there has been little research into the way people in prison are cared for and supported at the end of life. This study is part of a larger research project undertaken in Scotland. This project sought to explore palliative and end of life care in Scotland’s prisons, incorporating the perspectives of people in prison, their family members, prison officers, and prison healthcare staff. It also aimed to establish the roles played by community hospices in supporting prisons to provide palliative care, and to identify knowledge of and barriers and facilitators to palliative and end of life care in prison staff. The first step in this project – a rapid review of recent literature^11^ – has already been published, and this article will outline the findings of a qualitative study which focussed on the role of community hospices in supporting prisons to provide palliative care.

### Aim

The aim of this article is to describe the roles Scottish hospices have adopted to support prisons to provide palliative care, and to discuss the international relevance of these findings in addressing the knowledge gap around community hospices supporting people in prison.

## Methods

### Study design

This study employed a qualitative descriptive approach. As outlined by Sandelowski,^18,19^ qualitative description is a suitable approach when a straight description of the phenomena of interest is required.

### Population

The target population were Scottish adult hospices. A hospice was defined in this context as a specialist palliative care unit which provided both inpatient and outpatient care. Settings where palliative care was provided but not as the primary function, such as individual wards within larger hospitals or care homes which also provided palliative care were excluded. One representative was sought from each hospice who could provide information on any involvement the hospice had with prisons. The chief executive officer (CEO) was deemed to be an appropriate person to provide this information or to nominate someone within their organisation to participate.

### Sample

17 Scottish adult hospices as defined above were identified through the Scottish Partnership for Palliative Care.

### Recruitment

Emails outlining the purpose of the study, the questions to be asked and the proposed duration of the interviews were sent to the CEO of each hospice. They were also provided with contact information for the research team, and advised of their rights with regard to confidentiality and their right to withdraw from the study. Calls were arranged at the convenience of the interviewee, and prior to the start of the recorded interview, information about the study was read to the participant to ensure that they were able to provide informed verbal consent. Ethical approval for the study was obtained from the University of Glasgow’s College of Medical, Veterinary and Life Sciences Ethics Committee (Project number: 200180051; February 2019).

### Data collection

Semi-structured telephone interviews were conducted between February and May 2019, with three main questions to guide the conversation (Table 1). Interviewees were advised that the interview would take approximately 15 min, although some were longer (at the interviewee’s discretion, and with their consent). CM conducted all interviews. The calls were audio recorded, transcribed verbatim, and transcripts were imported into NVivo 12.^20^

**Table 1.** Telephone interview guide questions.

No.	Question
1.	Does your hospice have any links with a local prison?
2.	Has your hospice provided any advice or consultation to a local prison?
3.	Has your hospice had any prisoners as inpatients over the last 24 months?

### Data analysis

Data were analysed using Framework analysis.^21^ Framework analysis was originally developed for applied policy research, and has been used successfully in palliative care research.^22^ It is not tied to any particular philosophical paradigm.^23,24^ This study employs ontological realism with an interpretivist epistemology. The assumption is that there is an external reality, imperfect access to which can only be negotiated through human or social constructs; in this case through dialogue.

**Figure 1.**
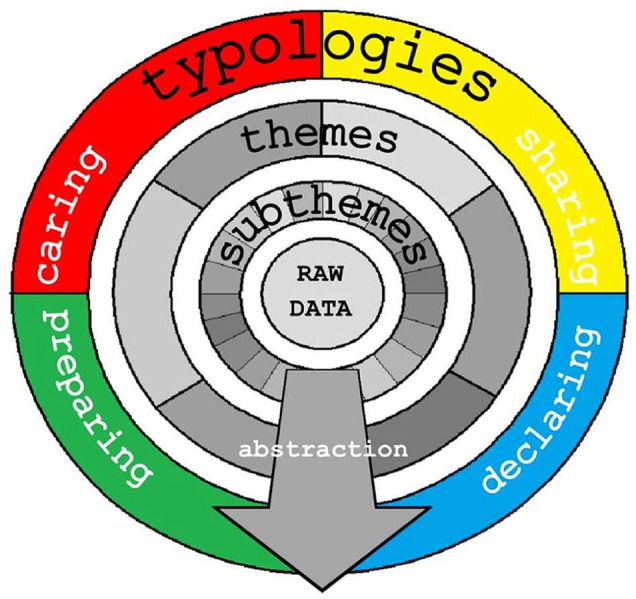
Conceptual map of framework matrix showing key themes.

Framework involves five stages: (1) familiarisation with the data, (2) identifying a thematic framework, (3) indexing the data using the thematic framework, (4) summarising the data on framework matrices or charts, and (5) mapping and interpretation of the data.^21^ One researcher (CM) conducted line-by-line inductive indexing of a sample of transcripts, and developed the thematic framework based on a combination of early themes and a priori factors related to the research question. The framework was then applied to another sample of transcripts before being further refined. Both researchers (CM and BJ) then agreed a final framework before it was systematically applied to the whole dataset. CM summarised and charted the data on framework matrices. Framework matrices allow for data abstraction to occur while remaining close to the raw data.^21^ Data were abstracted until typologies describing the roles adopted by hospices to support prisons were arrived at. This process is summarised in Figure 1. Moving with ease between raw data and more abstract concepts was seen to be a particular strength of Framework in this study. This allowed the researchers to ensure that the indexing, categories and final typologies provided a straight and useful description of the phenomena of interest,^18^ as was the aim of this study.

This study is reported in line with the consolidated criteria for reporting qualitative research (COREQ).^25^

## Results

All (*N* = 17) hospices participated. Various roles were represented (Table 2).

**Table 2.** Participants by job role.

Participant role	Number
Chief executive officer	2
Other managerial roles	8
Senior doctor	5
Senior registered nurse	2
*Total (n)*	*17*

The ways that hospices interact with prisons can be described using four simple typologies: *caring, sharing, declaring* and *preparing*. A summary of the activities which were categorised under each typology can be found in Table 3. Hospices employed different combinations of these roles, ranging from those who declared an openness to supporting prisons, to those who demonstrated all four types of behaviour. Table 4 outlines which hospices employed which roles. The different combinations of behaviours employed, can also be described using seven complex typologies, as can be seen in Table 4. These complex typologies help to show the variation in the levels of engagement demonstrated by hospices. However, for the purpose of describing the roles played by hospices the remainder of this article will focus on the four simple typologies of *caring, sharing, preparing* and *declaring.*

**Table 3.** Types of support provided by hospices to prisons.

Caring	• Providing direct care at the hospice to people who have been compassionately released from prison
	• Providing direct care at the hospice to people who are under escort by custody officers
	• Providing direct care to people who are still in prison (visiting the prison)
	• Consulting with prison staff in relation to decisions about compassionate release
Sharing	• Providing formal education sessions to prison healthcare and custodial staff
	• Holding sessions with people in prison and prison staff to promote conversations about death and dying
	• Providing telephone support to local prisons, as part of a service available to all clinical staff in the local area
	• Supporting prison psychiatry and chaplaincy to provide spiritual support to people who are dying in prison
	• Supporting prison psychiatry and chaplaincy to provide bereavement support to incarcerated relatives of hospice patients
	• Supporting prison healthcare staff with anticipatory care planning and introducing/maintaining palliative care registers
Preparing	• Initiating meetings with prison staff to gain an understanding of the prison environment and the palliative care needs of people in prison
	• Arranging for hospice staff to visit the prison to learn about the prison environment and the palliative care needs of people in prison
	• Arranging staff education sessions at the hospice featuring external speakers involved in custodial care and palliative care in prisons
	• Introducing and developing tele-mentoring systems to facilitate communication and knowledge-sharing about palliative care in prisons
Declaring	• Stating that they are open to the idea of supporting prisons to provide palliative care
	• Supporting people in prison to visit relatives who are inpatients at the hospice
	• Involving people in prison in work programmes to support rehabilitation, such as tending to hospice gardens

**Table 4.** Simple and complex typologies of behaviour.

Hospice	Simple typologies				Complex typologies
	Caring	Sharing	Preparing	Declaring	
H1					Carer/Sharer/Preparer/Declarer 3 Hospices
H2					
H3					
H4					Carer/Sharer/Declarer 1 Hospice
H5					Carer/Preparer/Declarer 1 Hospice
H6					Sharer/Preparer/Declarer 5 Hospices
H7					
H8					
H9					
H10					
H11					Sharer/Declarer 2 Hospices
H12					
H13					Preparer/Declarer 2 Hospices
H14					
H15					Declarer Only 3 Hospices
H16					
H17					
**Totals**	**5**	**11**	**11**	**17**	7 Combinations

Five (30%) hospices were engaged in *caring*, meaning that they had provided direct care to people either in prison or on their release. Eleven (65%) hospices were engaged in *sharing*, meaning that they had provided advice and support to prisons and prison healthcare teams to support prisoners with palliative care needs. The eleven (65%) hospices who demonstrated *preparing* were in the process of either developing or improving existing services to help support prisons. Finally, all seventeen hospices were classed as *declaring*, which means that they had demonstrated an openness to the idea of supporting prisons.

### Declaring

*Declaring* describes a hospice announcing their willingness to engage with prisons in order to provide palliative care. This also included demonstrations of willingness in the hospice’s past behaviours, such as engaging people in prison in work volunteer programmes, or supporting them to visit relatives who were hospice inpatients.

All hospices demonstrated *declaring.* Declarations of openness to caring for people in prison, and reflections on the problem of an ageing prison population were common. However, several hospices also described how they had accommodated people involved in the criminal justice system, either as visiting relatives of hospice patients, or through volunteer roles (such as maintaining the hospice gardens) designed to support their reintegration to society outside of prison. This was often framed as evidence of the hospice’s openness to the idea of supporting prisons. Some hospices were proactive in seeking out patients’ relatives who were in prison to facilitate visitation, and took pride in doing so:

*. . .we do everything we possibly can to make sure that it’s as neutral an environment as possible and that, you know, that person is accepted in just the way that any other family members visiting would be. [H14]*

However, some hospices also noted the risk of people in prison inadvertently gaining disproportionate access to specialist palliative care services due to a lack of adequate generalist palliative care services in prison. One participant stated:

*. . .I suppose, it’s just trying to work out where do the hospices sit I suppose in terms of what you might traditionally class as more specialist palliative care provision. I think by definition. . . being in the prison makes them more complex.*

*. . .one of the patients we’ve been involved with, had they not been in prison it wouldn’t necessarily have been somebody you think would have usually been referred to us, or you might have seen briefly and discharged. [H2]*

### Preparing

*Preparing* describes the process of hospices preparing to deliver care to prisons or preparing to improve upon their current methods of care delivery. In contrast with *sharing*, which describes hospices sharing their own knowledge and expertise, *preparing* involves the hospices learning from the prison and their own experiences.

One way in which hospices prepared to provide care was by building good relationships with their local prisons. Relationships ranged from tenuous, ad-hoc arrangements, to formal arrangements with regular meetings and collaborative approaches to providing care. Hospices and prisons established and nurtured these relationships through meetings, visitation between sites, and developing an understanding of each other’s roles. One hospice detailed a well-attended series of visits they had organised with their local prison:

*. . .the staff would go in and maybe groups of 12 or 15 and would be introduced to the prison, get a presentation from [a senior member of prison staff] about contacts, ageing populations, prisons not fit for purpose. . . how does our work meet your work, and then a tour of the prison, seeing the healthcare centre, seeing all the different cells. . . [H1]*

Other hospices brought in external speakers to deliver education sessions to hospice staff. Some hospices also discussed the use of innovative new tele-mentoring systems to help them communicate with each other and with the prisons, and were reflecting upon the practicalities of introducing this technology in future.

### Sharing

*Sharing* describes hospices sharing knowledge, advice and expertise with the prisons or other hospices.

The most common way that hospices described sharing their knowledge with prisons was on an as-required basis, and over the phone. Most stated that this is no different to how they would provide advice to any other clinician who requested it:

*We have a 24-hour, seven day a week on-call service for professionals. So, any practitioner, nurse or doctor can phone to get senior medical advice about any patient irrespective of where that person is, so that would include the prison. [H12]*

Some, however, employed a more structured approach to sharing knowledge and advice. This included promoting events to get people in prison to talk about death and dying, and delivering education sessions to prison staff. While some reported largely positive responses from the prison staff, others encountered a degree of resistance:

*I think some of the prison officers felt that they weren’t there to do the health element of it, and death and dying wasn’t really their business. However, that’s the point of the training programme, was saying death and dying is everybody’s business. [H8]*

Hospices were also involved in supporting prison psychiatry and chaplaincy teams to provide spiritual support to those who were dying, and to relatives in prison of hospice patients who had died. Others were involved in sharing good examples of palliative care practices into the prison, such as anticipatory care planning and the introduction of palliative care registers.

### Caring

*Caring* describes hospices actively providing palliative care to people in prison, either at the hospice or in the prison.

Mechanisms exist in Scotland, the UK and in many other countries, which allow people who have a terminal condition and limited prognosis to be released from prison to a more appropriate setting at the end of their life. The process of applying for compassionate release was viewed as a complex procedure by the hospices; one that must be initiated early and considered on a case-by-case basis. A large proportion of the ageing prison population are incarcerated for sexual offences, and the restrictions associated with this group can impact upon decisions related to compassionate release:

*. . .you know, we have children here all the time, so say if they came and they were compassionate released, they wouldn’t be under guard. If they were still mobile, they would have to be confined to a room, and are we going to exert those constraints? So a lot of the time we have said as a hospice, we could probably only support this person to be compassionately released in the hospice setting when actually they are bed bound and unable to get out of bed [H1]*

Even when the hospice felt confident that they could accommodate the individual, the likelihood of them being granted compassionate release in a timely manner (or at all) was perceived to be small. More than one hospice suggested that the high-profile case of the ‘Lockerbie bomber’ Abdelbaset al-Megrahi, who died almost 3 years after being compassionately released from a Scottish prison in 2009, had a negative impact on decisions.

However, compassionate release is not the only way for a someone to receive care at a hospice. In the UK, they can also be escorted to the hospice by custody officers, who remain with them so long as they are in lawful custody. Some felt that the presence of custody officers could make hospice staff and other patients uncomfortable, with clinicians worrying about the impact it had on confidentiality. Others, meanwhile, reflected on the fact that the officers themselves were probably not prepared for or expecting to accompany a dying person on shift, and that their wellbeing should be considered. Careful planning of the placement and movement of escorted persons within the hospice was recommended by those who had experience of doing so.

The importance of managing and supporting hospice staff who are going to be caring for people involved in the criminal justice system was also discussed. Approaches varied between hospices, but all were centred around the importance of preparing staff properly. Some hospices had engaged in educating their staff either through formal education sessions, or through arranging visits to the local prison. Both approaches were seen to have a positive impact on the way staff felt about providing care. There was a great deal of discussion around the level of information required about the individual by staff, particularly in relation to their crimes. Most felt that it was limited to the minimum required to guarantee staff safety (such as whether there is history of violent or sexual crimes):

*. . .they wanted to know. . . was the offender a sex offender, is what they wanted to know. When I said to them no, they weren’t really that bothered any more. [H5]*

Others, meanwhile, felt that it would be impossible to control whether staff searched for further information about crimes online, and the most important thing would be to ensure that the person’s confidentiality was respected in the workplace.

However, not all care is delivered within the hospice. Some hospices delivered outpatient care to people in prison. Some debate surrounded which approach was the best, with time often being the deciding factor:

*. . .you’d need a couple of hours really to go and see the one patient, whereas if they were coming to see you in clinic it would be half an hour [H2]*

Clearing security and moving about within the prison was seen to be a deciding factor, yet it was not the only barrier to the provision of care. The physical environment and the strict regime which is required to maintain security within a prison can impact on care delivery. The administration of controlled medications or use of specialist equipment such as hospital beds were cited as activities which were challenged by the environment and regime. For some people, prison may be the closest place they have to a home, and these barriers can be sufficient to prevent them from being able to die there. One clinician provided an example:

*. . .she had said, you know, this is like my family now. You know, so her friends there, they become like family. And she’s being supported by these people rather than by her family, because of the situation. So, yes I would think from what she was saying, reading between the lines, that that would have been her preferred choice. And I suppose potentially it is her home, so to speak. . .*

*. . .now, there wasn’t an option for her to die in the prison. . . you wouldn’t have been able to fit all the equipment we needed into her room. . . it would have been a fire risk, because you couldn’t get a bed through the door if a fire was to start in the prison. So, it was just no, no, she wouldn’t have been able to die there. [H3]*

*

In summary, hospices engage with local prisons in several ways, including declaring their willingness to do so, preparing to provide care, sharing their expertise, and actively providing care to those at the end of life.

## Discussion

Recent research has described in detail prison-based hospices in the United States, the elements that contribute to their success, and the complex role played by inmate hospice volunteers.^5,26–31^ The typology presented here describes the ways that hospices in Scotland engage with prisons in Scotland. Yet we believe that this typology is of international relevance, particularly to the many countries who have not established hospices within the prison walls. The ongoing mapping exercise being conducted by the EAPC taskforce^12^ suggests that many countries are similar to Scotland in that they do not operate prison-based hospices. A series of literature reviews have identified that most research published on palliative care in prisons comes from the United States,^1,11,14,32^ where hospice care is frequently delivered within the prison walls by a dedicated prison hospice.^33^ In this way, the evidence base does not fully represent the ways that different nations are contending with the global problem of people ageing and dying while in prison. This study is one of the first to describe an alternative model, one which relies on close collaboration between multiple agencies and individuals to balance the palliative care needs of the person at the end of life with the necessary functions of a criminal justice system.

It is not possible to conclude from this study whether the support provided by hospices is adequate to meet the needs of those who may die in Scotland’s prisons; further research will be required to evaluate this. However, hospice support for prisons in Scotland is at an early stage in its development, and ageing and dying while incarcerated is still a common fear for many people in prison,^11^ partly due to worries about the perceived inadequacy of palliative care provision.^34^ Unmet healthcare needs are a common feature for those who are in prison, both during and at the end of life.^35^

Prior to this study, it was known that 30% of Scottish hospices provide support to prisons.^36^ In this study, 30% (*n* = 5) of hospices were indeed involved in providing direct care, yet it is reasonable to argue that the 65% (*n* = 11) of hospices described as *sharing* also provide support in the form of sharing expertise. Incorporating those who were involved in *preparing* to care suggests that as many as 82% (*n* = 14) of hospices are currently involved with prisons in some capacity. However, there are differences in the degree of involvement demonstrated between hospices. Those who were actively involved in *caring* described a complex process of relationship building, staff preparation, effective communication, and clear oversight of the transfer process, all of which were necessary to facilitate care delivery. While *declaring* sometimes involved facilitating visitation to the hospice or involvement in work programmes, it could also be demonstrated simply by announcing a willingness to engage with the prison population.

Geographical proximity may be a factor in this variation, yet distance between prisons and hospices does not entirely remove the potential for hospices to support people on their release from prison. People may be compassionately released to a geographically distant area to the prison they were liberated from, depending on where they were resident before prison or where their family live. This was a driving factor in some of the more distant hospices’ attempts to build relationships with prisons. However, all hospices who were involved in *caring* were physically close to the prisons they were involved with. Similarly, grouping people in prisons with certain characteristics may cause geographical variations in the demand for hospice support. Countries including Australia, the Czech Republic, France and Slovakia have prisons which are only for those serving long sentences,^12^ and are, therefore, more likely to develop palliative care needs during their time in prison. In England and Wales there are specific prisons for people convicted of sexual offences,^12^ another population who – partly due to the growing number of convictions for historic sexual offences – are often older and more likely to require palliative care. Our experience in Scotland indicates that there are some prison populations more likely to need palliative care due to the clustering of these characteristics in specific prisons.

There are differences and similarities between the way different countries support those with palliative care needs in prison. In a 2002 discussion paper, Dawes advocated for a combination of improved use of existing compassionate release policies and in-prison hospice care in Australia.^37^ Yet almost 20 years later there are no dedicated palliative care services in Australian prisons, although some states and territories do utilise external palliative care services to provide care within the prison.^12^ Recent research by Panozzo and colleagues in Australia also identified constraints and tensions in providing end of life care to hospitalised people experiencing incarceration, similar to those discussed in this study.^38^ Participants in a 2017 study by Chassagne and colleagues also noted the shortcomings of French prisons as places to care for someone who is dying and advocated for better use of compassionate release policies.^2^ Other options in France include access to hospital wards within the prison which are affiliated with local hospitals, or high-security wards set within existing community hospitals. Yet, there is no specific system (such as prison hospices) in place for palliative care, and compassionate release is seen as the preferred option.^2^ In their 2017 study, Handtke and colleagues found that older people in Switzerland’s prisons had high expectations of being compassionately released from what they perceived to be an unsuitable environment when they approached the end of life – although these expectations were in contrast with their experiences of others seeking compassionate release.^39^

Despite variation in the way that different countries care for those who are living with palliative care needs in their prisons, there are aspects of the problem which transcend borders, and are also evidenced in this study. Firstly, the prison environment presents challenges to the provision of palliative care. Secondly, that compassionate release is a favourable option when appropriate, and desirable to the person in prison. Compassionate release is an important mechanism for palliative care providers to consider, and the issue of people being released to be cared for at the hospice featured heavily in the interviews. Sexual and violent offences were discussed as key factors to consider in relation to decisions surrounding compassionate release, yet there are many more; a comprehensive discussion of which can be found in a recent content analysis of US policies by Holland et al.^40^ While compassionate release policies vary between countries, research from across the globe^6,39–42^ indicates that it is not applied for or secured as frequently as it could be. Post-release support is an important factor in decisions related to compassionate release,^40,43^ and hospices should consider whether they are adequately prepared to care for someone on their release. This extends not only to ensuring that the security and confidentiality of the individual and other patients can be guaranteed, but also that staff are prepared for and supported throughout the experience.

Much has been written about the conflict between custody and care in this context; this is not a conflict between the priorities of hospices and the priorities of prisons. The purpose of a prison is far removed from that of a hospice,^44^ yet both owe a duty of care to people with specialist palliative care needs in prison. Marti et al.^45^ argue that rather than conflicting or colliding, care and custody overlap and blur when prisons support those who are dying. Our data suggests that hospices are also adopting an approach to balancing care and custody which requires an understanding of both specialist palliative care and the demands of custodial environments and the criminal justice system in which they operate. Achieving this balance is dependent on both prisons and hospices becoming familiar with the way each other operates. Hospices sharing their expertise on specialist palliative care and developing an understanding of the complexities of prison life are taking steps to reconcile these competing priorities. Similarly, those who are open to the prospect of supporting people in prison and those developing services for this population are responding to the growing need to extend the reach of palliative care.^46^

Collaboration between prisons and hospices, and the development of a mutual understanding of each other’s roles will be essential to the extension of palliative care into custodial environments. Particularly amongst those who were engaged in providing direct care to people in prison, there was evidence of a high-level understanding of the complexities of the prison setting and the challenges associated with this context, echoing what is already known about the challenges faced by prison healthcare staff and specialist palliative care providers.^44^ It is important that this knowledge is shared not only within the palliative care community, but also with other prisons where support is needed – this is particularly important when these prisons and hospices have limited experience supporting people in prison at the end of life. This study found great variation in the level of engagement individual hospices have with individual prisons, and this is to be expected. Prisons which are situated in the same locality as a hospice, who house a large proportion of older persons, and a large proportion of people serving lengthy or indeterminate sentences are much more likely to have cause to engage with their local hospice. For other hospices, providing support over the telephone on an as-required basis may be sufficient to meet the needs of their local prison population. Yet the quality of care someone receives should not be affected by these geographical variations. Our data shows that sharing knowledge and communication between prisons and hospices across Scotland is common, and this will be a key factor in ensuring that expertise is shared not just between local dyads of prisons and hospices, but on a national level. Establishing robust networks of prisons and hospices on a national level will aid the development of services for this population.

## Limitations

The use of relatively short telephone interviews may have limited the depth that could be achieved when compared with lengthier face-to-face interviews. However, the hospices are spread across Scotland and conducting face-to-face interviews within the allotted time would not have been feasible. The decision to keep interviews brief was taken on the basis that the study sought to recruit CEOs and other individuals who were unlikely to be able to allocate a large amount of time to the interview. It was envisioned that the response rate may be higher when only requesting 15 min. Those who wished to speak for longer were welcomed to do so.

Our recruitment strategy sought individuals with the organisational oversight required to provide an overview of the hospice’s links to prisons. As such, the sample consists only of CEOs, senior doctors, senior nurses and managers. To better represent the multidisciplinary nature of palliative care, the perspectives of other hospice colleagues such as social workers, chaplains, counsellors and volunteers should be included in future research.

There is also a risk of social desirability bias in studies where participants self-report attitudes or behaviours. This was considered during data analysis, and the typology was primarily based on the actions undertaken by hospices, as opposed to attitudes and perceptions. The exception to this is *declaring*.

Finally, the perspectives of people in prison are absent from this study, and should be included in further research. This study comprises one part of a larger project which has sought to include the voices of incarcerated persons, the findings of which will be disseminated in the near future.

## Conclusion

The support provided by hospices to prisons extends beyond direct care, and many hospices are also involved in sharing their expertise in relation to specialist palliative care. Others are developing services to meet the demand of this population, with the assistance of prisons and those involved in custodial care. For the many countries who have not adopted prison-based hospices, effective collaboration between prisons and community hospices on a national level will be required to meet the needs of the growing number of people in prison who require palliative care.
